# Characterization and Model Validation for Large Format Chopped Fiber, Foamed, Composite Structures Made from Recycled Olefin Based Polymers

**DOI:** 10.3390/polym12061371

**Published:** 2020-06-18

**Authors:** Daniel P. Pulipati, David A. Jack

**Affiliations:** Department of Mechanical Engineering, Baylor University, Waco, TX 76706, USA; Daniel_Pulipati@baylor.edu

**Keywords:** structure-property relations, modeling, micro-mechanics, foams, recycling, polyolefins

## Abstract

The purpose of this research is to predict the material performance of large format foamed core composite structures, such as crossties or structural timbers, using only constitutive properties. These structures are fabricated from recycled post-consumer/post-industrial waste composed of High-Density Polyethylene (HDPE) and Glass Filled Polypropylene (GFPP). A technical challenge in predicting the final part performance is the mathematical correlation between the microstructural variations and the macroscopic responses as a function of fiber aspect ratio, cell density, and constitutive properties of the polymer blend. The structures investigated have a dense and consolidated outer shell and a closed cell foamed core. The non-linear shell and the foamed core material properties are analyzed with micromechanics models, and the reference stress of the shell and core is predicted using a modified Rule of Mixtures model. The predicted properties are used as the inputs for a Finite Element Analysis (FEA) model, and the computational results are compared to experimental four-point bend test results for sixteen samples performed on a 120-kip compression stage. The results show that the mean of the characterized deflections from the four-point bend tests did not show any variations for an isotropic and transversely isotropic model using a linear analysis. This model was then extended to a non-linear analysis using the Ramberg–Osgood model to predict the full crosstie four-point bend test behavior. The FEA model results show a deviation of 2.45 kN compared to the experimental variation of 3.58 kN between the samples measured.

## 1. Introduction

**Figure 1:**
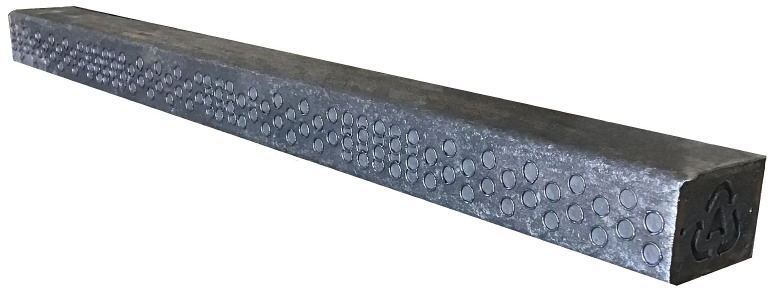
Example of a composite crosstie.

Polymers, specifically polyolefins, are widely used in most household and industrial applications. However, many researchers have found there are negative effects of high consumption and waste generation (see, e.g., [1,2,3]). Therefore, finding new ways to reuse recycled polymers and polymer matrix composites is essential to reduce the amount of waste. In the past decade, large format recycled polymer composites have found applications in composite structures such as short bridges, construction mats, railroad crossties (sleepers), and so on. The use of recycled polymer composite crossties has increased, and thousands of composite crossties have been in service throughout the world [4]. A typical polymer matrix composite crosstie is shown in Figure 1. Wooden crossties are being replaced with composite crossties made of recycled High-Density Polyethylene (HDPE) and Glass Filled Polypropylene (GFPP) because wooden ties have a shorter expected lifespan and require chemically toxic preservatives such as creosote to increase their lifespan. There is a desire for an alternative material because creosote is a known environmental hazard with various health issues, and it has the potential to contaminate the water and soil when used in crossties [4]. Researchers have extensively reviewed alternative materials for crossties and noted fiber polymer matrix composites as a reasonable alternative solution (see, e.g., [5,6,7]). Polymer matrix composite crossties have outstanding durability, ease of manufacture, and high structural performance, and they also provide flexibility for in situ crosstie replacements to replace hardwood timber crossties [5].

Short fiber composites are used extensively in a wide variety of manufacturing methods, such as injection molding, extrusion molding, compression molding, and recently in additive manufacturing. The presence of fibers in such applications has been studied by various researchers to understand the effect of fibers on the overall material properties of the composite material. Researchers have extensively studied and developed micromechanics models to predict an overall anisotropic stiffness of a unidirectional fiber reinforced composite material (see, e.g., [8,9,10,11]). Tandon and Weng [12] modeled an iterative method using the constitutive elastic properties of the matrix and the fiber to predict the transversely isotropic stiffness of a unidirectional composite material by extending Mori and Tanaka’s method [10]. Tucker and Liang [13] studied the related micromechanics models for fiber reinforced composites with aligned fibers of uniform length and linearly elastic isotropic mechanical properties and they have suggested the Mori–Tanka model to be reasonably accurate over the range of aspect ratios used in the present study. Tucker and Liang also provided a modification of Tandon and Weng’s explicit model by decoupling the plane-strain bulk modulus and major Poisson ratios and used a direct solver without iteration, the full form of which is fully expressed in Zhang [14].

To model the stiffness properties of a chopped fiber composite, the geometric aspect ratio of the individual fibers is essential. Therefore, an accurate understanding of the length and diameter distribution of the as manufactured composite is necessary in the prediction of the stiffness properties. The reduction in the fiber length distribution depends on process parameters such as back pressure, speed of revolution, barrel temperature and the polymer melt shear rates [15]. In quantifying the fiber length distribution, it was shown in [16] that over 800 fiber samples, properly selected from within a sample set, are required to achieve an error less than 5%. Fiber measurements can be biased based on improper sampling on the edges of the sample area, fiber breakage during sample preparation, and improper discrimination for very short and very long fibers, the details of which are expounded upon in Section 2.2. The weight-based aspect ratio gives preference to the contribution of the longer fibers and is more appropriate for use in micromechanical predictions, whereas the number based aspect ratio gives equal weight to fully intact fibers and the multiple sub-structures of a fiber that is broken during manufacturing [16].

**Figure 2:**
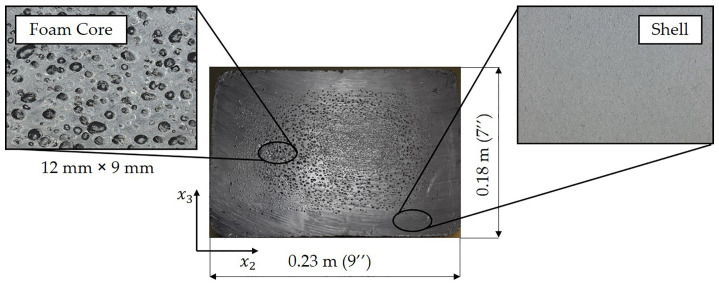
Cross-section showing the shell and foamed core regions of the crosstie.

The use of foaming agents during manufacturing results in a non-uniform closed cell structure using current manufacturing techniques as indicated in Figure 2. The overall behavior of the closed cell composite depends on the shape, volume fraction, cell size distribution and material properties surrounding the closed cells [17]. Micromechanics models to predict the effective stiffness for closed cell foams have been studied extensively by various researchers (see, e.g., [18,19,20,21]). McLaughlin [22] and Farber and Farris [19] have developed methods to predict shear and bulk modulus of a polymer composite with spherical inclusions based on a differential scheme. Wang et al. [23] developed a two-phase model, along with three-phase composite models for spherical inclusions based on the Mori–Tanka method and obtained numerical results for normalized moduli. Zhang et al. [24] studied high density polyethylene closed cell homogenous spherical foams prepared by compression molding, and they compared various models to calculate the elastic modulus. They concluded that the differential scheme and empirical equations using power-law and the square power-law provide reasonable estimates of the stiffness over a cell volume fraction ranging from 0 to 55%, a range that encompasses the cell density considered in this research.

Lo et al. [25] studied the effective stiffness for a transversely isotropic closed cell PVC foam using a unit cell representation. This is done by substituting an apparent volume fraction for the actual resin volume fraction. Gong et al. [26] studied the impact properties of Polypropylene (PP) foamed composites at different temperatures (−80 to −20 ${}^{\circ }$C and −20 to 20 ${}^{\circ }$C) and showed the impact properties of foamed PP are greater than the unfoamed PP in the lower temperature range −80 to −20 ${}^{\circ }$C. Therefore, it can be noted that material properties of the foamed composites vary based on temperature. In this present study, all the tests were performed at room temperature.

Various researchers have studied recycled composite crossties/plastic lumber and its non-linearity to understand the impact variations as a function of feedstock material on the resulting mechanical properties and failure behavior. Martinez-Guerrero [27] studied the effects of viscoelastic creep over a range of temperatures for recycled polymer crossties based on various blends of HDPE with polyethylene terephthalate and polystyrene and concluded that the plastic lumber can be considered as elastic under short term loading. Bajracharya et al. [28] studied plastic solid wastes consisting of HDPE, PP and LDPE (Low Density Polyethylene) by manufacturing samples for coupon and full-scale testing and measured their strengths based on tensile, flexure, shear, and compression testing. They predicted the non-linear flexure behavior of the polymer specimens by using coupon testing results and applying them to a finite element model (FEM) and compared the FEM results to a Fiber Model Analysis (FMA). In a subsequent paper, Bajracharya et al. [29] extended the study by using glass fibers to the plastic solid wastes and used the modified rule of mixtures and FMA to predict the strength and elastic properties of glass fiber reinforced plastic solid composites by using constants to account for orientation and fiber length factor. Lofty et al. [4] studied the performance and flexural behavior of composite crossties made of HDPE using an FEM with model inputs based on tensile tests from dog-bone specimen obtained from the manufactured crosstie.

This paper presents a methodology for modeling the performance of a foamed composite crosstie using only constitutive properties and compares the predicted results with experimental observations. The novelty of this approach is that the model predictions begin at the constitutive level and includes the following: variation in fiber length, cell distribution, fiber packing density, fiber orientation alignment, and the constitutive behavior of the fibers and the matrix. Model inputs for the constitutive behavior of the matrix are obtained from tensile specimens of the neat polymer prior to blending. Experimental testing is performed to quantify the fiber length, which is a model parameter. The experimental validation of the predicted structural performance is accomplished using four-point bend results of full-scale crossties. The experimental and predicted results that will be presented for the stiffness and the corresponding linear deflection model are shown to be within 2% of each other. To analyze the non-linear behavior of the tie, the elastic properties obtained from the fiber orientation modeling for the shell and the foamed core are used along with reference stress predictions at 0.2% strain. The constructed model is then used to study the sensitivity to model parameter variations, providing insight for improving the final processed product performance. The results presented in this paper show that the most sensitive parameter is the volume fraction of the glass fibers followed by the volume fraction of the closed cell foams.

## 2. Methodology

Thermoplastics have the benefit of being reheated above their melt temperature and remolded into a new shape. In the present paper, we focus on the use of Polypropylene (PP) and Polyethylene (PE) due to their extensive use in industry. They comprise close to 50% of all recycled polymers (see, e.g., [30]), in part due to their ease of sorting based on their low density. From a structural perspective, the modulus values for PP and PE are considered low in the range of 0.5–2 GPa based on the blend. To increase the stiffness of the polymer, glass fibers are commonly used as a reinforcement because they are inexpensive and easy to manufacture. To obtain various material and thermal properties of the input materials for the micromechanical study, tensile tests were performed based on ASTM D638-03 for the HDPE and the crosstie sections. A detailed fiber length analysis was performed to measure the aspect ratio which served as an input to the micromechanical model. To incorporate the material properties based on closed cells, foam density calculations were performed. To demonstrate the approach, four-point bent tests were conducted and compared with a full finite element simulation.

### 2.1. Manufacturing and Testing

The composite structures studied in this paper are a blend of HDPE and GFPP formed into a crosstie (sleeper) for use in the rail industry. The crosstie is manufactured using an extrusion process where the recycled HDPE and GFPP are blended with carbon black and a blowing agent. The compound, which consists of ~80% HDPE, ~19% of GFPP, carbon black and the blowing agent, is mixed and melted under temperatures in excess of 200 ${}^{\circ }$C and is pressurized using a screw assembly to fill a mold cavity. This blowing agent forms a dense outer region called the shell region with few if any cells. The inner region is composed of a series of closed cells created by the blowing agent and is termed the core. The foamed core region has varying cell sizes with a cell density of approximately 26 ± 2.9%. Results will show that the resulting structural performance varies as a function of the cell size and density. The recycled HDPE and GFPP are from residential and commercial waste streams, and come from a moderately controlled feedstock, due to the nature of the recycling industry. These variations are briefly studied in the present work and incorporated within the present model to understand the impact on final part performance.

To characterize the input tensile modulus values for use in the modeling efforts of this work, a batch of the HDPE feedstock without fibers was tested to obtain the modulus values. Dog bone shaped tensile bars were made using a micro 12 cc injection molding machine (Xplore Instruments BV, Sittard, Netherlands). HDPE was melted at 195 ${}^{\circ }$C, and the mold was held at room temperature. The fill and holding pressure were each 12 bar. The specimens were then tensile tested using a 100 series universal testing machine (TestResources. Inc, Shakopee, MN, USA) following ASTM D638-03. The load-displacement curve obtained from the test was then used to calculate the elastic modulus using the initial loading curve slope regression techniques based on the initial linear elastic region. This displacement was measured using an extensometer. For the HDPE, the tensile tests were performed at a constant crosshead speed of 50 mm/min. Six samples were tested to capture the mean elastic modulus and the associated standard deviation of the neat polymer system.

Based on the stress-strain results obtained from the recycled HDPE tensile tests, the reference stress of HDPE was measured at 0.2% reference strain. Even though the matrix material properties consist of both HDPE and PP, the stress of HDPE is the dominant contributing factor for the composite crosstie. When observed for a blend of up to 60% HDPE and 40% PP, the stress completely shifts towards the material with the lower stress, in this case the HDPE [31]. As the PP constituent is ~10% of the total composite, the effect of PP on stress can be ignored. Based on the stress of the HDPE, the stress of the composite $\sigma_{yc}$ can be predicted using the modified rule of mixtures (see, e.g., [29]),

(1) $$\begin{array}{c} \sigma_{yc}\;=\;\chi_{1}\chi_{2}v_{f}\sigma_{f}+\left(1-v_{f}\right)\sigma_{m} \end{array}$$

where, $\chi_{1}$ is a constant taken to be 1/6 for randomly oriented fibers [32], $\chi_{2}$ is the fiber length factor, $v_{f}$ is the volume fraction of the fibers, $\sigma_{f}$ is the tensile stress of the fibers, $\sigma_{m}$ is the reference stress of the matrix. $\chi_{2}$ is based on the ultimate stress of the fiber $\sigma_{f}$, the weight average fiber length $L_{w}$, critical fiber length $L_{c}$, the interfacial shear strength between fibers and matrix τ, and the radius of the fibers $r_{f}$ [33] and is given as

(2) $$\begin{array}{c} \begin{array}{c} \chi_{2}=\frac{L_{w}}{2L_{c}}\\ L_{c}\;=\;\frac{r_{f}\sigma_{f}}{\tau } \end{array} \end{array}$$

Since the closed cell foams induced by the blowing agent reduces the overall stress of the foamed core, it is necessary to predict the reference stress of the core based on the normalized foam density *f*, which is given by (see, e.g., [34])

(3) $$\begin{array}{c} f\;=\;(1-\frac{\rho_{foam}}{\rho_{shell}}) \end{array}$$

where, $\rho_{foam}$ is the bulk density of the foamed composite core and $\rho_{shell}$ is the density of the solid shell composite. To predict the normalized stress of the foamed core $\sigma_{yf}$, the reference stress of the shell is used with a square-power relationship as suggested by Zhang et al. [35] and is given as

(4) $$\begin{array}{c} \sigma_{yf}=\sigma_{yc}{\left(1-f\right)}^{2} \end{array}$$

**Figure 3:**
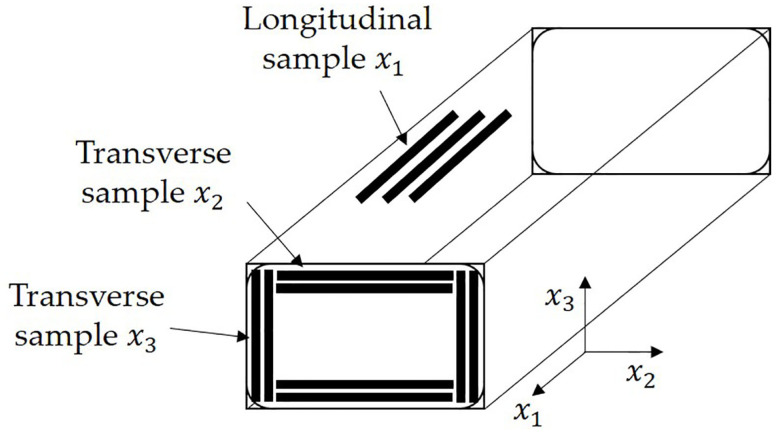
Samples taken from various sections of the crosstie to measure the longitudinal and transverse tensile modulus.

To characterize the as manufactured spatially varying anisotropic moduli of the composite, samples were sectioned from the manufactured composite. Samples were cut from a section of a manufactured crosstie using a miter saw to measure the tensile modulus along vertical and horizontal transverse directions and the longitudinal direction for the shell region. Seven samples from each of the transverse directions, $x_{2}$ and $x_{3}$, and eight samples from the longitudinal direction $x_{1}$, as shown in Figure 3, were tested. The crosshead speed was reduced to 5 mm/min because the fiber reinforced composite fails at a lower strain than that of the neat HDPE. Each sample was tested until failure, and the corresponding tensile modulus of the tie in the three directions were recorded.

### 2.2. Fiber Aspect Ratio

**Figure 4:**
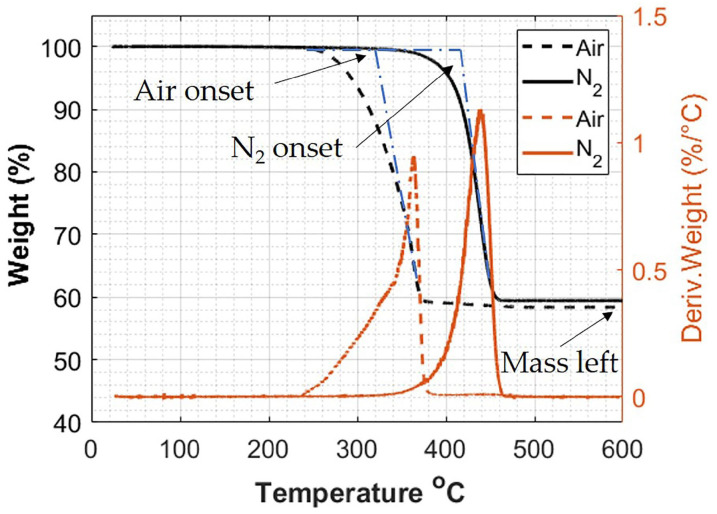
Thermogravimetric Analysis comparison showing the decomposition of glass filled polypropylene in Air and N_2_.

The manufactured crosstie performance demonstrates a subtle sensitivity to variations in the fiber modulus but is highly sensitive to changes in the fiber aspect ratio. Of interest, then, is the resulting fiber breakage caused by the high shears during the manufacturing processes. Thus, the as manufactured aspect ratio is of interest and an approach similar to that of [16] will be performed. A Q50 Thermogravimetric Analyzer (TA Instruments, New Castle, DE, USA) was used to observe the decomposition of GFPP in the presence of air and nitrogen, and typical results are shown in Figure 4.

The onset degradation temperature of PP was measured to be 325 ${}^{\circ }$C in the presence of air and 415 ${}^{\circ }$C in N_2_. In addition, the Thermogravimetric Analysis (TGA) results indicate that there is a complete burn off of the resin system with no loss of fiber mass at 600 ${}^{\circ }$C in either air or N_2_: there is no change in the weight % after the decomposition step. This was a potential concern, as it has been shown that other fibers, such as Carbon and Kevlar, oxidize at elevated temperatures in the presence of air [16]. Of additional interest, the graphs shown in Figure 4 confirm that there is no change in the decomposition at the elevated temperatures, regardless of the presence of air or N_2_, and the fibers make up ~59% of the mass of the polypropylene fiber composite.

To collect fibers for the length study, a 2 cm^3^ block of the solid shell region was sectioned and heated to 600 ${}^{\circ }$C in the presence of air to fully decompose the polymer matrix, leaving only fibers and some ash residue in the crucible (see, e.g., [23]). The fibers were collected in a 200 mL beaker and suspended in distilled and purified water. The water suspension was then sonicated using a Branson 450 digital sonifier (Branson Ultrasonics, Danbury, CT, USA) with a disruptor horn at 20% amplitude until the fibers were detangled. Approximately 15 mL of fiber suspension was collected in a glass petri dish and placed in an air convection furnace at 75 ${}^{\circ }$C until all the water was evaporated.

**Figure 5:**
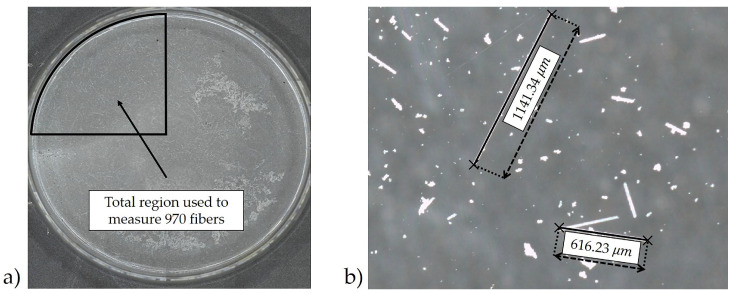
Steps for glass fiber measurements to determine the aspect ratio of the fibers (**a**) stitched image showing fibers collected in a petri dish (**b**) example fiber length measurements.

**Figure 6:**
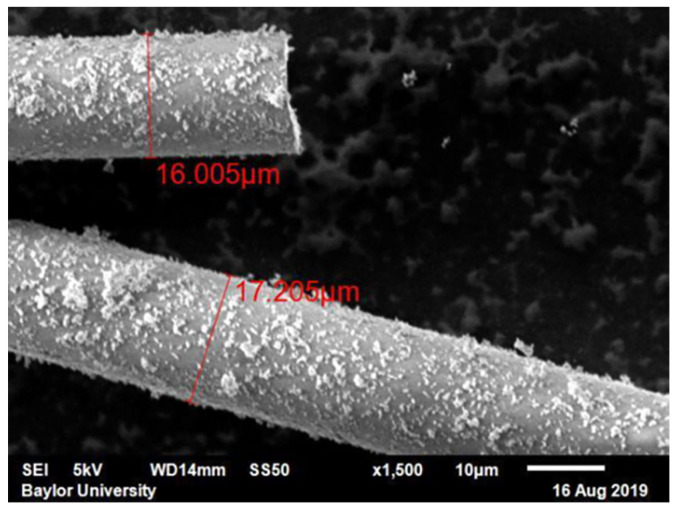
Example of diameter measurements taken from a Scanning Electron Microscope.

The resulting fiber residue was imaged using a VR-3100 3D Measurement Macroscope (Keyence Corporation of America, Itasca, IL, USA), and multiple macroscopic images were stitched together as shown in Figure 5a. The length of every single fiber within the marked section of the petri dish image was measured once regardless of the length. An example fiber length measurement from 2 fibers is shown in Figure 5b. Diameter measurements were obtained using a JSM-6610LV Scanning Electron Microscope (JEOL Ltd., Tokyo, Japan), and a typical image is shown in Figure 6. The number average and weight average aspect ratio of the fibers were calculated from 970 samples for the length and 50 samples for the diameter. The choice of 970 fibers was made upon the suggestion by Sharma et al. [16] where due to the asymmetry of the length distribution they found that over 800 fibers were necessary to well characterize the length distribution. Conversely, only 50 fibers were used in the diameter measurements due to the well behaved statistical nature of the diameter distribution as it was well represented by a normal distribution. The full results along with the statistical justification is given in Section 3.2.

### 2.3. Modeling the Solid Shell and the Foamed Core

**Figure 7:**
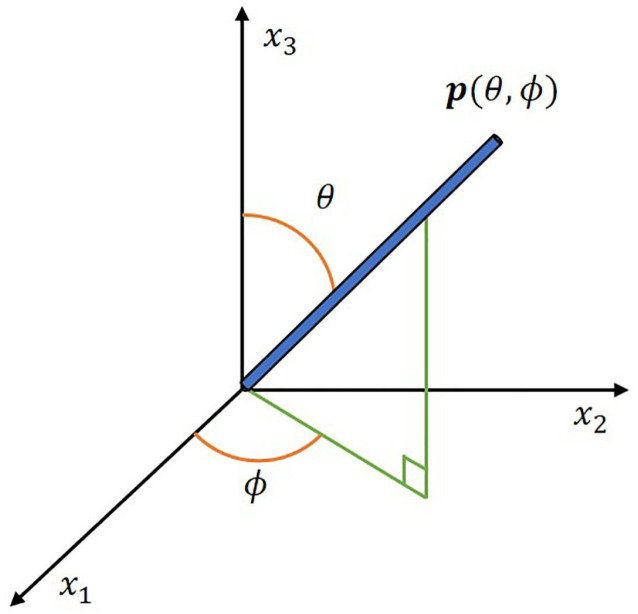
Unit vector representation.

The drastic difference in the length scales between the 100 μm~1 mm fibers and a 2.62 m tie preclude a model from having a mesh over individual fibers. Thus, a continuous probability distribution function ψ(θ,ϕ) of fiber orientation is used similar to the description given in [36], where the orientation of an individual fiber can be represented by a unit vector as seen in Figure 7 and defined as

(5) $$\begin{array}{c} p=\left[\begin{array}{c} sin\theta \;cos\phi \\ sin\theta \;sin\phi \\ cos\theta \end{array}\right] \end{array}$$

The cylindrical fiber ends are indistinguishable from each other, thus the probability of finding a fiber along the angles θ and ϕ must be equal to finding a fiber in the opposite direction, therefore,

(6) $$\begin{array}{c} \psi (\theta ,\phi )\;=\;\psi (\pi -\theta ,\pi +\phi )\;\;\;or\;\;\;\psi (p)\;=\;\psi (-p). \end{array}$$

The fiber orientation distribution function description remains too cumbersome for modeling an entire composite. To address this issue, Advani and Tucker [36] developed a modeling technique using orientation tensors to express the average orientation of the fiber suspension. The second order orientation tensor $a_{ij}$ and the fourth-order orientation tensor $a_{ijkl}$ are defined as

(7) $$\begin{array}{c} a_{ij}=\int_{S}p_{i}p_{j}\psi \left(p\right)dS,\;\;\;\;\;a_{ijkl}=\int_{S}p_{i}p_{j}p_{k}p_{l}\psi \left(p\right)dS \end{array}$$

where, S is the surface of a unit sphere. The orientation tensor is symmetric by construction. For example, $a_{ij}\;=\;a_{ji}$, and $a_{ijkl}\;=\;a_{klij}\;=\;a_{jikl}\;=\;a_{ilkj}\;=\;\cdot \cdot \cdot$.

The integral over the unit sphere of the fiber orientation distribution function is 1. From this property, the trace may be shown to be 1, i.e., $a_{11}+a_{22}+a_{33}$ = 1. Similarly, the higher order orientation tensor fully contains the lower ordered tensor, i.e., $a_{ij11}+a_{ij22}+a_{ij33}\;=\;a_{ij}$. In this paper, the orthotropic fitted (ORT) closure method developed by VerWeyst and Tucker [37] is used to approximate the fourth order orientation tensor $a_{ijkl}$ in terms of the second-order orientation tensor $a_{ij}$. The ORT closure has been demonstrated by Montgomery-Smith [38] as one of the most accurate approximations.

The current study uses the Advani and Tucker [36] homogenization approach to calculate the anisotropic stiffness based on the fiber orientation distribution obtained and the underlying unidirectional composite stiffness tensor ${\bar{C}}_{ijkl}$. In the present study, we use the approach presented by Tandon–Weng [12] to predict the elastic stiffness of a unidirectional composite from the matrix properties, fiber properties and the fiber aspect ratio. The average material properties for the range of Glass Fiber and Polypropylene are taken similar to those in [39]. Once the unidirectional stiffness tensor ${\bar{C}}_{ijkl}$ is calculated, the local effective anisotropic stiffness tensor $\langle C_{ijkl}\rangle$ is obtained using the equation,

(8) $$\begin{array}{cc} \langle C_{ijkl}\rangle = & B_{1}a_{ijkl}+B_{2}\left(a_{ij}\delta_{kl}+a_{kl}\delta_{ij}\right)+B_{3}(a_{ik}\delta_{jl}+a_{il}\delta_{jk}+a_{jl}\delta_{ik}\\ & +a_{jk}\delta_{il})+B_{4}\left(\delta_{ij}\delta_{kl}\right)+B_{5}\left(\delta_{ik}\delta_{jl}+\delta_{il}\delta_{jk}\right) \end{array}$$

where $\delta_{ij}$ is the Kronecker delta, and the $B_{i}$ terms are expressed in terms of the underlying unidirectional stiffness tensor as (see, e.g., [36]),

(9) $$\begin{array}{c} \begin{array}{cccccc} B_{1} & = & {\bar{C}}_{1111}+{\bar{C}}_{2222}-2{\bar{C}}_{1122}-4{\bar{C}}_{1212} & B_{4} & = & {\bar{C}}_{2233}\\ B_{2} & = & {\bar{C}}_{1122}-{\bar{C}}_{2233} & B_{5} & = & \frac{1}{2}\left({\bar{C}}_{2222}-{\bar{C}}_{2233}\right)\\ B_{3} & = & {\bar{C}}_{1212}+\frac{1}{2}\left({\bar{C}}_{2233}-2{\bar{C}}_{2222}\right) \end{array} \end{array}$$

For the present study, two orientation states are considered. The first is a fully random orientation state given as $a_{11}=a_{22}=a_{33}=1/3$. The second is a representative transversely isotropic state given as $a_{11}=1/2,a_{22}=a_{33}=1/4$. This latter state matches the directional nature observed in the structural testing caused by fiber alignment due to shearing [23]. Future work will utilize orientation measurements similar to those of Vélez-García [40] to quantify the spatially varying orientation state, but this is beyond the present scope. Once the fourth order orientation tensor is calculated for $a_{ij}$ using the ORT closure, Equations (8) and (9) are used to obtain the fourth order stiffness tensor. Then the fourth order compliance tensor can be calculated using the inverse relationship $<S_{ijkl}>$ = $<C_{ijkl}{>}^{-1}$. The resulting effective elastic moduli can be calculated using the relationships

(10) $$\begin{array}{c} E_{11}=\frac{1}{S_{1111}},\;\;E_{22}=\frac{1}{S_{2222}},\;\;E_{33}=\frac{1}{S_{3333}} \end{array}$$

The closed cells in the foamed core depicted in Figure 2 will reduce the effective modulus relative to that of outer solid shell region. The density of the foamed core composite is measured by weighing six blocks of foamed composite assuming that the closed cells are spatially uniform in shape and size. In this paper, we assume the material properties within the foamed region to be isotropic, spherical, uniform in size, and uniform in spatial distribution. Using the $<C_{ijkl}>$ obtained from the solid composite from Equation (8), the engineering elastic constants can be calculated. Zhang et al. [24] reviewed a series of popular closed cell foamed models, and their results showed that the differential scheme power law model given by McLaughlin [22] yields acceptable results for the density of the present system. This model will be used to predict the closed cell foam core elastic modulus, which is given as

(11) $$\begin{array}{c} E_{f}\;=\;E_{11}{\left(1-f\right)}^{n} \end{array}$$

where, $E_{f}$ is the elastic modulus of the normalized foamed core and *n* is taken to be 1.96 based on the Poisson’s ratio of 0.44 for the crosstie [24].

### 2.4. Four-Point Bend Testing

**Figure 8:**
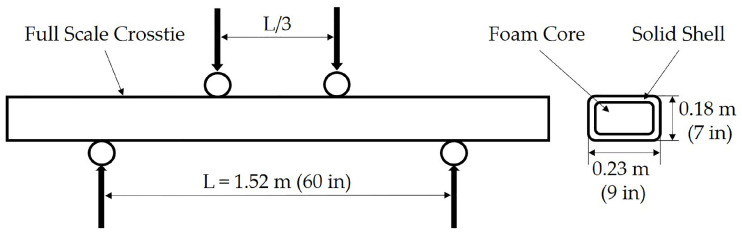
Representative four-point bend test (dimensions for experimental and FEA study).

The manufactured crossties were tested using four-point bend tests for Modulus of Rupture (MOR) and Modulus of Elasticity (MOE) based on ASTM D6109-13 [41], as depicted in Figure 8 and only the MOE is reported in the present study. The span for the 2.62 m (103.2 inch) long tie was 1.52 m (60 inch). The four-point bend test allows for a uniform load distribution within the interior span and is a preferred test method recommended by the American Railway Engineering and Maintenance-of-Way Association (AREMA). A custom Tinius Olsen universal mechanical test bench that has a capacity of 533 kN (120 kip) was used for the four-point bend flexural testing at a control rate of 12.7 cm/min (5 in/min) and was loaded until failure. In total, sixteen samples were used in this study.

**Figure 9:**
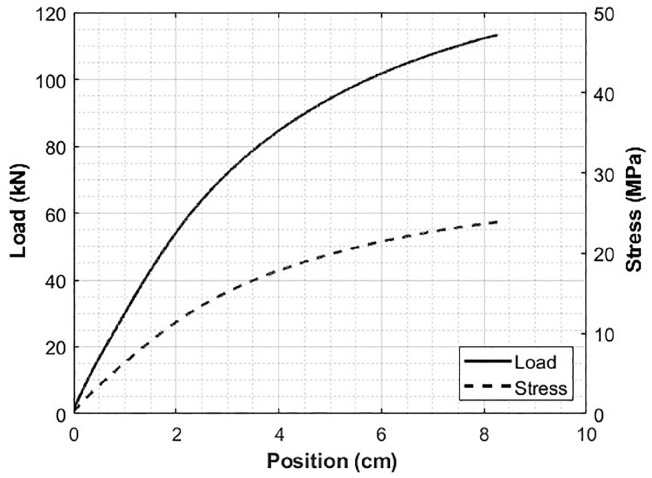
Example load-deflection curve of a crosstie from a four-point bend test.

The applied load and deflection of the crossties were measured during testing for each of the crossties. For each of the crossties tested, the exterior dimensions were measured along with the shell wall thickness. A typical load-deflection curve is shown in Figure 9. Using Equations given in ASTM D6109, the stress *S*, and strain *r*, are calculated as,

(12) $$\begin{array}{c} S\;=\;PL/bd^{2}\\ r\;=\;4.70Dd/L^{2} \end{array}$$

where *P* is the total applied load on beam, *L* is the total span length on the beam, *b* is the width of the beam, *d* is the depth of the beam and *D* is the measured deflection [41].

The calculated MOE represents the equivalent geometric material properties, and it does not differentiate the contribution in either the shell or the foamed core regions. The MOE is calculated by measuring the linear portion of the load-deflection curve and dividing the flexural stress measured at any point in the linear region. It is worth noting that the crossties tested in this study meet and exceed the requirements for crossties based on AREMA requirements for both MOE and MOR as shown in [4]. Even though the load-deflection curve is non-linear as shown in Figure 9, the first study in the region of interest for the crosstie is taken to be the initial linear region. Within the linear region, the slope of the load-deflection curve is used in calculating the MOE, and the average results are presented in Section 3.4. This linear model was then compared to the FEA results modeled using isotropic and transversely isotropic models to investigate the sensitivity of fiber orientation to the final part behavior. The linear study was then extended to a non-linear isotropic model to predict the full force deflection curve, as seen in the tested crossties.

### 2.5. Finite Element Analysis

A finite element analysis of the full crosstie subjected to a four-point bend test was performed to validate the modulus values. The model uses inputs based on the constitutive properties of the polymer and fibers. There were distinct regions for the solid shell and the foamed core. The dimensions of the crosstie were 0.229 m × 0.178 m × 2.62 m (9” × 7” × 103.2”). As there was some variation in the shell thickness between crossties, the shell thickness was measured on every crosstie, and an average value from the specimen of 0.81” was taken for the modeling efforts. An average deflection was calculated for each of the applied forces, and the resulting slope of the load deflection curve was then compared to the initial slope of the physical load deflection curve.

To model the full non-linear behavior of the crosstie, a method suggested by Ramberg-Osgood [42] was used and is shown as,

(13) $$\begin{array}{c} \varepsilon \left(x\right)=\frac{\sigma (x)}{E}+\varepsilon_{ref}{\left(\frac{\sigma (x)}{\sigma_{ref}}\right)}^{n} \end{array}$$

where ε(x) is the strain at every stress σ(x), and $\varepsilon_{ref}$ is taken at the 0.2% reference strain obtained from the predicted reference stress $\sigma_{ref}$ of the shell using Equation (1) and of the core using Equation (4). The reference strain was chosen based on the recommendation of COMSOL (Version 5.4, COMSOL Inc., Burlington, MA, USA). The FEA results were similar with other selected reference strains, for example, 0.3% or 0.5% strain. The reference stress of the shell is obtained from the modified rule of mixtures and the reference stress of the tested HDPE. The core reference stress is predicted using the square power law equation based on the predicted shell composite reference stress. *E* is the elastic modulus and *n* is the stress exponent. The stress exponent *n* is calculated by fitting the stress and strain parameters from the tensile tests of the recycled HDPE to Equation (13) and is taken as 4 for all modeling efforts.

## 3. Results

### 3.1. Tensile Testing

Tensile tests conforming to ASTM D638-03 were performed to calculate the elastic modulus of raw material HDPE and the manufactured tie. The elastic modulus values from 6 samples are presented in Table 1. The $x_{2}$ and $x_{3}$ axis represents the cross section of the tie, and the $x_{1}$ direction represents the direction of the mold filling as depicted in Figure 3. In the initial stages of the research, the crosstie’s elastic modulus was assumed to be isotropic, but based on the modulus values seen in Table 1, there is a clear directional bias to the modulus values. The modulus along $x_{2}$ and $x_{3}$, transverse to the mold filling axis, are seen to be statistically similar.

### 3.2. Fiber Aspect Ratio

**Figure 10:**
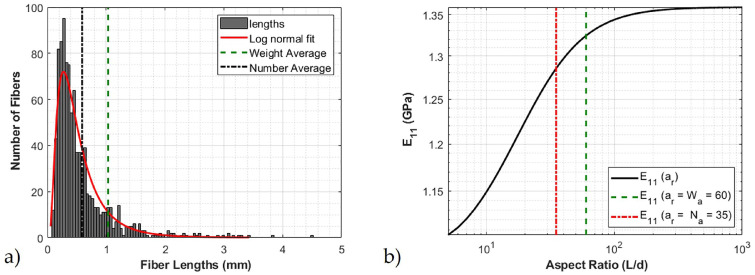
Glass fiber characteristics (**a**) Fiber length distribution (**b**) Effect of aspect ratio on the elastic modulus.

The radius of the fiber was measured to be 8.56 μm ± 0.57 μm with a skewness of −0.19. The low absolute value relative to 1 of the skewness indicates that the radius distribution is well represented by a normal distribution. Thus, there will be little statistical change in the reported results when one increases the number count in the data set and 50 samples well represents the overall nature of the distribution. From the fiber length measurements, a weight average and number average aspect ratio were calculated. The number average fiber length was measured to be 0.583 mm, and the weight average fiber length was measured to be 1.004 mm, with a skewness from the data of 6.7. A skewness greater than 1 indicates that a distribution is poorly represented by a normal distribution, and as the skewness increases the number of samples required in the data set to properly capture the statistical nature of the distribution also must increase. The histogram of the 970 fiber length measurements is shown in Figure 10a. The number average and weight aspect ratio were measured to be 35 and 60, respectively, which are depicted in Figure 10a. The weight average aspect ratio was used in the constitutive modeling based on the recommendation of Sharma et al. [16]. It can also be noticed that the fiber lengths appear to be reasonably represented by a log normal distribution. A short study is presented in Figure 10b for the modulus of the glass-fiber system with the properties of the matrix from Table 2 as a function of increasing aspect ratio from 1 to 100 using the Tandon and Weng model [12]. It can be observed from Figure 10b the choice in number average and weight average makes a 3% difference in the investigated composite. The small difference in modulus is due to the low volume fraction of the glass fibers in the overall composite and would be more pronounced for higher fiber packing densities.

### 3.3. Material Properties Prediction

The constitutive properties of the matrix and fibers are given in Table 2 and are used to create the predicted isotropic modulus for a random orientation state and transversely isotropic modulus for a representative transversely isotropic orientation state as presented in Table 3. The overall matrix Young’s modulus was calculated using the rule of mixtures to account for the modulus of HDPE and PP. The isotropic orientation state is given as $a_{11}$ = $a_{22}$ = $a_{33}$ = 1/3 with all other $a_{ij}$ values being zero, and the transversely isotropic orientation state $a_{11}$ = 1/2, $a_{22}$ = $a_{33}$ = 1/4 with all other $a_{ij}$ values being zero. These orientation states, along with the constitutive properties of Table 2, are used in Equation (8) to achieve the effective stiffness tensor. Observe from Table 3 that the predicted modulus of an isotropic orientation state is less than the measured longitudinal moduli for the transversely isotropic orientation state. Comparing the model moduli of Table 1, it is clear that the transversely isotropic orientation state is more appropriate, with an error less than 2.5% for each of the moduli.

Using the predicted composite material properties and the measured volume fraction of the closed cells, the foamed closed cell material properties were predicted based on the micromechanics models presented in Section 2.3. Six different density measurements of the solid composite and the closed cell foamed composite were taken and a volume fraction of the closed cell was calculated to be 26 ± 2.9% of the solid shell composite.

**Figure 11:**
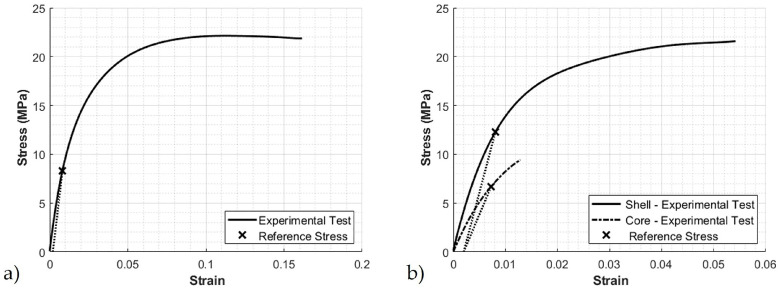
Reference stress at 0.2% reference strain obtained from (**a**) stress-strain curves from HDPE tensile tests (**b**) stress-strain curves for the shell and core.

To calculate the stress of the solid shell and the core, the stress measured from tensile testing of the recycled HDPE material is used at a 0.2% reference strain. The stress measured was 8.3 MPa, as shown in Figure 11a. The interfacial shear stress τ was taken as 15 MPa, and the glass fiber strength $\sigma_{f}$ was taken as 3450 MPa, as suggested in [29]. This produced a predicted stress of the shell and core of 12.48 MPa and 6.83 MPa, respectively. These values were then compared to the experimental reference stress measured from the tensile test results of the shell and core and are shown in Figure 11b. Notice the predicted reference stress for the shell and core follow the trends seen in the tensile tests of the shell and core samples given in Table 4.

### 3.4. Finite Element Analysis

Two studies were performed to analyze the sensitivity of isotropic and transversely isotropic material properties for the domain depicted in Figure 8 and discussed in Section 2.3. The first study was the isotropic properties from the micromechanics results, and the second study was the transversely isotropic results for the moduli and Poisson’s ratio, presented in Table 3.

**Figure 12:**
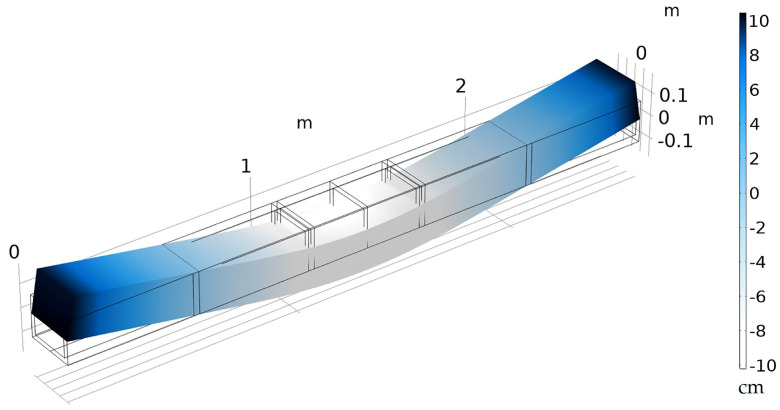
Representative load-deflection simulation with finite element analysis in COMSOL.

**Figure 13:**
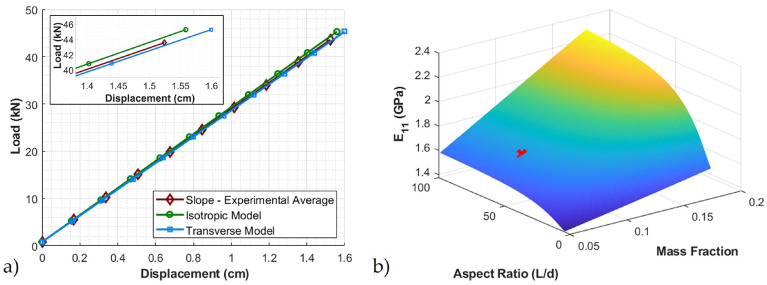
(**a**) Model validation of linear elastic load-deflection with finite element analysis (**b**) effect of elastic modulus with change in aspect ratio and volume fraction of fibers.

Figure 12 shows the 4-point bend test simulation with the deformation for a 111 kN (25,000 lb) force. From Figure 12, it is clear that the largest deflection occurs at the center. The experimental results and the model predictions for maximum deflection as a function of loading are presented in Figure 13a for a loading up to 45 kN for both the isotropic and transversely isotropic models. The results from the isotropic and transversely isotropic model results do not deviate substantially in this study as the change in the longitudinal modulus values are not substantially different. The slope of isotropic and transversely isotropic models is calculated and compared to the slope calculated from the experimental testing results. The MOE calculated from FEA is 1.67 GPa and 1.63 GPa, for isotropic and transversely isotropic modulus, respectively, which is quite similar to the experimental observations of an MOE of 1.64 GPa.

The model can be easily extended to investigate the input of final part performance based upon changes in the material parameter inputs. Specifically, in the present work we studied the effect of aspect ratio on the final part material properties and coupled that with the effect of change in the mass fraction of the fibers. The results of this study can be seen in Figure 13b. For the given aspect ratio and mass fraction of the glass fibers, the elastic modulus given is shown with a marker ‘x’. The mass fraction is varied from 5–20% and the aspect ratio is varied from 1–100. From this study, if the MOE of the crosstie was needed to be increased, one could do this by increasing the volume fraction of the aspect ratio by a predictable amount. As seen in Figure 10b, with the increase in aspect ratio, there is only a small increase in $E_{11}$, whereas the increase in $E_{11}$ is far more sensitive to a change in the fiber volume fraction.

To extend the linear FEA model and study the non-linear behavior of the crosstie, the Ramberg–Osgood method given in the COMSOL non-linear structural module was used. The Ramberg–Osgood method uses three parameters, which are, reference stress, reference strain, and Young’s modulus, to fit the stress-strain curves.

**Figure 14:**
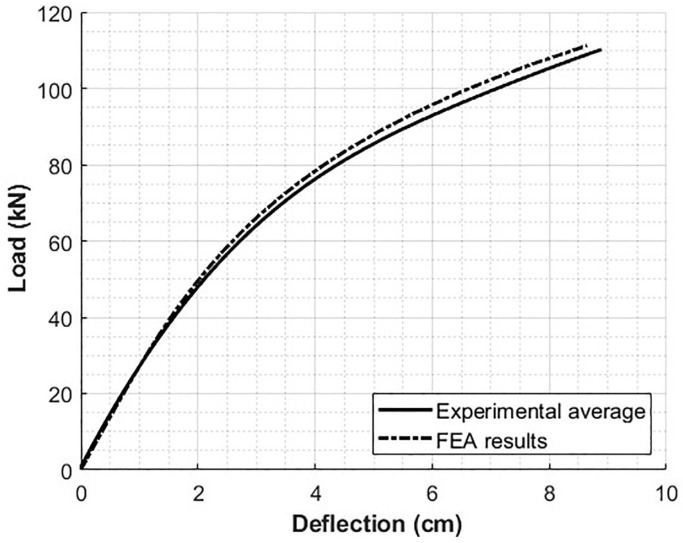
Non-linear FEA model using the Ramberg–Osgood model in comparison with the experimental four-point bend test average.

Therefore, as seen in Table 4, the Young’s modulus values, which were obtained from micromechanics models along with the reference stress predicted at 0.2% reference strain, were used and the results are shown in Figure 14. It can be seen that the finite element analysis results follow a similar trend when compared to the experimental average results.

To quantify the effectiveness and have a reasonable agreement between the model and experimental results, an error defined as the difference in the average experimental load-deflection curve and the FEA load-deflection curve, is defined as,

(14) $$\begin{array}{c} Err_{model}\;=\;\frac{\int_{0}^{d_{max}}\left|L_{exp}\left(x\right)-L_{model}\left(x\right)\right|dx}{d_{max}} \end{array}$$

where the error is normalized using the maximum deflection, and in this study is calculated at $d_{max}$ = 8 cm. This result is then compared to the error between each individual experimental test and the average load-deflection curve is defined as

(15) $$\begin{array}{c} Err_{exp,i}\;=\;\frac{\int_{0}^{d_{max}}\left|L_{exp}\left(x\right)-L_{i}\left(x\right)\right|dx}{d_{max}} \end{array}$$

The experimental error is then averaged over the range of samples as,

(16) $$\begin{array}{c} Err_{exp}\;=\;\frac{\Sigma Err_{exp,i}}{No.\;of\;samples} \end{array}$$

The experimental average of the load-deflection curve for the crosstie up to 111 kN (25,000 lbf) yields an $Err_{model}$ of 2.45 kN. This value can be interpreted as the average error between the model prediction and the average of the experimental results. This error is then compared to $Err_{exp}$, which is the sample to sample variability and is 3.58 kN. Therefore, the model prediction as presented is within the bounds of the experimental variability.

## 4. Conclusions

In this paper, a composite structure made of recycled HDPE, GFPP, carbon black, and foaming agents using extrusion molding is discussed. This structure is a solid composite in the outer shell with a foamed inner core. Based on the material properties of the raw materials and fiber aspect ratio, the engineering material properties of a spatially varying composite structure is predicted using various fiber orientation and micromechanical models. Samples were cut from the crosstie along the cross section and in the longitudinal direction, and tensile tests were performed on the sectioned samples. The predicted transversely isotropic modulus values agree with the measured elastic modulus values.

A finite element analysis simulation was developed to measure the load-deflection of the crosstie based on the ASTM D6109-13 four-point bend test. Both isotropic and transversely isotropic material behaviors were modeled based on the predicted properties. Sixteen crosstie test specimens were tested, and an experimental average load-deflection was measured. All the ties were tested until failure and the modulus of elasticity (MOE) of the tested crossties exceeded the minimum requirements based on AREMA. The MOE of the developed model and the experimental four-point bend test were within 2% error for both the isotropic and transversely isotropic micromechanical models.

The linear FEA model was then extended to predict the full non-linear load-deflection curve using the Ramberg–Osgood model. It was seen that there is an agreement to the experimental four-point bend tests and the non-linear FEA results with an error bounded by 2.45 kN against the mean load-deflection curves, which themselves had a deviation bounded by 3.58 kN between samples. Lastly, the usefulness of the developed model was demonstrated by studying variations in the final part properties as a function of fiber aspect ratio and volume fraction of glass fibers. This method can be further modified to include localized varying material properties in the core due to varying cell sizes and distributions. Finite element models can also be implemented to account for both transient analysis due to the viscoelastic nature of the polymer matrix and spatial variations due to local variability within the foamed core. This technique can be easily extended to model over multiple crossties under in-situ loading conditions within the transportation industry.

## Figures and Tables

**Table 1:** Experimental tensile testing results for HDPE and tie samples.

Material	Solid Axis Direction	Tensile Modulus (GPa)	Std Dev (GPa)
HDPE		1.23	0.06
Tie	$x_{1}$	1.99	0.24
Tie	$x_{2}$	1.62	0.16
Tie	$x_{3}$	1.63	0.27

**Table 2:** Micromechanics input material properties.

Material Property	Value
Matrix modulus	1.29 GPa
Fiber modulus	70 GPa
Aspect ratio	60
Volume fraction	3.33%
Poisson’s ratio of the fiber	0.23
Poisson’s ratio of the matrix	0.45

**Table 3:** Modulus values from micromechanical predictions used in FEA modeling.

Model	Solid Axis Direction	Material Properties
Isotropic Modulus	$x_{1}-x_{2}-x_{3}$	1.73 (GPa)
Transversely Isotropic Modulus	$x_{1}$	1.95 (GPa)
Transversely Isotropic Modulus	$x_{2}$	1.66 (GPa)
Transversely Isotropic Modulus	$x_{3}$	1.66 (GPa)
Core Modulus		0.96 (GPa)
Poisson’s ratio		0.44

**Table 4:** Material inputs for the non-linear FEA model.

Material	Property	Result
Shell	Tensile Modulus	1.73 (GPa)
	Reference Stress	12.48 (MPa)
	Reference Strain	0.002
Core	Tensile Modulus	0.96 (GPa)
	Reference Stress	6.83 (MPa)
	Reference Strain	0.002
